# FPGA-based RF interference reduction techniques for simultaneous PET–MRI

**DOI:** 10.1088/0031-9155/61/9/3500

**Published:** 2016-04-06

**Authors:** P Gebhardt, J Wehner, B Weissler, R Botnar, P K Marsden, V Schulz

**Affiliations:** 1 Division of Imaging Sciences and Biomedical Engineering, King’s College London, London WC2R 2LS, UK; 2 Department of Physics of Molecular Imaging Systems, Institute of Experimental Molecular Imaging, RWTH Aachen University, 52062 Aachen, Germany; 3 Philips Research Europe, 52066 Aachen, Germany; pierre.gebhardt@kcl.ac.uk; volkmar.schulz@pmi.rwth-aachen.de

**Keywords:** PET–MRI, RF interference reduction, MR compatibility, FPGA, digital, SiPM

## Abstract

The combination of positron emission tomography (PET) and magnetic resonance imaging (MRI) as a multi-modal imaging technique is considered very promising and powerful with regard to *in vivo* disease progression examination, therapy response monitoring and drug development. However, PET–MRI system design enabling simultaneous operation with unaffected intrinsic performance of both modalities is challenging. As one of the major issues, both the PET detectors and the MRI radio-frequency (RF) subsystem are exposed to electromagnetic (EM) interference, which may lead to PET and MRI signal-to-noise ratio (SNR) deteriorations. Early digitization of electronic PET signals within the MRI bore helps to preserve PET SNR, but occurs at the expense of increased amount of PET electronics inside the MRI and associated RF field emissions. This raises the likelihood of PET-related MRI interference by coupling into the MRI RF coil unwanted spurious signals considered as RF noise, as it degrades MRI SNR and results in MR image artefacts. RF shielding of PET detectors is a commonly used technique to reduce PET-related RF interferences, but can introduce eddy-current-related MRI disturbances and hinder the highest system integration. In this paper, we present RF interference reduction methods which rely on EM field coupling–decoupling principles of RF receive coils rather than suppressing emitted fields. By modifying clock frequencies and changing clock phase relations of digital circuits, the resulting RF field emission is optimised with regard to a lower field coupling into the MRI RF coil, thereby increasing the RF silence of PET detectors. Our methods are demonstrated by performing FPGA-based clock frequency and phase shifting of digital silicon photo-multipliers (dSiPMs) used in the PET modules of our MR-compatible *Hyperion II*^*D*^ PET insert. We present simulations and magnetic-field map scans visualising the impact of altered clock phase pattern on the spatial RF field distribution, followed by MRI noise and SNR scans performed with an operating PET module using different clock frequencies and phase patterns. The methods were implemented via firmware design changes without any hardware modifications. This introduces new means of flexibility by enabling adaptive RF interference reduction optimisations in the field, e.g. when using a PET insert with different MRI systems or when different MRI RF coil types are to be operated with the same PET detector.

## 1. Introduction

Multimodality imaging is nowadays an important part of clinical protocols whenever complementary imaging information of anatomical, functional and physiological types leads to improvements with regard to diagnosis and therapy. These include a better lesion detectability and localisation, improved patient response monitoring, optimisation and acceleration of imaging protocols, and enhancement of patient ease during imaging procedures (Townsend 2008b, Zaidi and Del Guerra 2011). Positron emission tomography (PET), one of the main imaging techniques used in nuclear medicine, is capable of visualising a wide range of metabolic processes at molecular level with the highest sensitivity while offering biomarker distribution quantification possibilities *in vivo* (Zaidi *et al* 2006, Kitson *et al* 2009). However, the downsides of PET are a relatively low spatial resolution (in the range of a few millimetres in case of clinical systems) and the fact that it delivers patho-physiological rather than anatomical information. With the introduction of single devices combining PET with computer tomography (CT) (Beyer *et al* 2000), patient studies undergone with PET–CT successfully demonstrated the benefit of combined PET–CT, particularly for cases of ambiguous or uncertain diagnostic situation, which could be clarified by the added value of CT imaging to precisely localise physiological lesions (Townsend 2008a).

Magnetic resonance imaging (MRI) is a modality enabling *in vivo* anatomy imaging at high resolutions. Compared to CT, it does not make use of ionising radiation, offers excellent soft-tissue contrast and provides functional information e.g. via spectroscopy, perfusion or diffusion imaging, thereby making hybrid PET–MRI highly desirable for e.g. paediatric, neurology and cardiovascular imaging in addition to oncology (Raylman *et al* 2007, Judenhofer *et al* 2008, Cherry 2009, Kwee *et al* 2010, Buchbender *et al* 2012, Catana *et al* 2012). When performed simultaneously, PET–MRI benefits from very accurate temporal and spatial image registration, thereby allowing us to increase the quantitative as well as qualitative accuracy of PET images by applying MRI-based motion correction of PET (Tsoumpas *et al* 2010, Buerger *et al* 2012, Catana *et al* 2012, Chun *et al* 2012), which is inherently not possible with PET–CT or sequential PET–MRI. Furthermore, patients who need to undergo both a PET and an MRI scan usually experience a long total imaging procedure when scans are done sequentially. Simultaneous PET–MRI benefits from shorter scan times compared to the sequential approach, which accelerates the clinical work flow and is of interest in terms of patient ease, particularly regarding paediatric, fragile and older patients.

The advantages and possibilities potentially offered by combined PET–MRI led numerous research institutions and companies during the last two decades to address the challenging task of integrating PET detectors into MRI systems with the aim of enabling truly simultaneous bi-modal image acquisition while keeping the intrinsic performance of PET and MRI subsystems unaffected. The latter is obtained by simultaneous operation of the two modalities without any unwanted, mutual interferences, which otherwise lead to image quality deterioration of PET as well as MRI.

These interferences, to be avoided by a careful PET–MRI device design, are (static and dynamic) magnetic as well as electromagnetic field related and can be classified as follows.

(i) MRI-related interferences capable of negatively affecting PET operation.
  (a) Strong, constant, magnetic MRI B_0_ field can cause electronic components to be disturbed (Hall effect affecting semiconductor devices such as digital temperature sensors) or to fail, e.g. coils equipped with ferrites saturating, Lorentz force application on electrons in photomultiplier tubes (PMTs).
  (b) Switching MRI gradient fields resulting in eddy current generation within conductors (cables and printed circuit board (PCB) traces) as well as conducting areas (surfaces), which may lead to electric signal disturbances and material heat-up.
  (c) Electromagnetic (EM) excitation fields in the radio-frequency (RF) range (RF fields for short), also known as the MRI system’s B_1_ field, which induce voltages and eddy currents in conducting materials such as device housing and shielding, but also cabling and PCB traces. In the latter two cases, electric signal disturbances may result.
(ii) PET-related interferences disturbing the MRI operation.
  (a) Materials of the PET detector with non-zero susceptibility (especially ferromagnetic components) causing distortions of the MRI B_0_ field homogeneity, which results in MR image impairment.
  (b) Potential MRI gradient field disturbances in a spatial and temporal sense caused by eddy currents originating from the PET detectors, which result in MRI spatial encoding perturbations.
  (c) RF fields emitted by PET detectors, which might couple into the MRI RF receive coil and generate disturbances related to the MRI signal-to-noise ratio (SNR). This leads to an MRI SNR reduction when assuming an unaltered MRI signal strength and consequently introduces a deterioration of MR image quality.

Compared to PMTs typically used for PET scintillation light detection, semiconductor-based avalanche photo-diodes (APDs) and silicon photo-multipliers (SiPMs) can be operated within strong magnetic fields, thereby making APDs and SiPMs very suitable for PET detectors to be operated within or in close proximity to an MRI bore (Vandenberghe and Marsden 2015). Since their introduction, numerous PET detector systems for PET–MRI based on APD (Judenhofer *et al* 2007, Solis *et al* 2008, Catana *et al* 2008, Delso *et al* 2011, Maramraju *et al* 2011, Vaska *et al* 2011, Kolb *et al* 2012) or SiPM technology (Hong *et al* 2012, Yamamoto *et al* 2012, Yoon *et al* 2012, Hong *et al* 2013, Levin *et al* 2013, Weissler *et al* 2014, Olcott *et al* 2015, Weissler *et al* 2015a) have been presented. In this case, the analogue APD/SiPM signals are either transmitted via copper-based, shielded cables or optical fibres from the MRI bore to the PET electronics residing outside the MRI bore to be digitized and processed (Judenhofer *et al* 2007, Maramraju *et al* 2011, Hong *et al* 2012, Kolb *et al* 2012, Yamamoto *et al* 2012, Yoon *et al* 2012, Hong *et al* 2013, Olcott *et al* 2015), or are digitized within the MRI bore, requiring more electronics to be moved towards or housed within the MRI (General Electric (2014), Solis *et al* 2008, Maramraju *et al* 2011, Vaska *et al* 2011, Weissler *et al* 2014, 2015a)(Vandenberghe and Marsden (2015), p. R140–1).

Analogue signals transmitted over a signal line with arbitrary length may alter regarding their original shape and intensity (amplitude) due to transmission-media-related attenuation and distortion, and the addition of undesired, disturbing signals along the transmission path. Unwanted signals adding to the original information signal by distorting it are referred to as *noise* (Ott 1988). In the case of electrical signalling, crosstalk of nearby (adjacent) signals as well as externally sourced electromagnetic fields irradiating signal lines are both causes of noise generation. When data are transmitted analogously, i.e. in the form of a signal with continuously varying values (frequency, amplitude), (pre-)amplifiers are usually used to increase the gain of signals e.g. originating from APDs or SiPMs. However, not only is the information signal amplified, but also the noise which adds to the signal during transmission towards the amplifier input, leading to decreased SNR. By using encoding to represent signal information digitally via discrete amplitude levels, undesired noise can be removed and its amplification overcome. For instance, when data are binary encoded, an amplitude level below a lower threshold is considered a logical ‘0’ and a level higher than an upper threshold means a logical ‘1’. If the interval between the thresholds is large enough compared to the noise levels distorting the actual information signal, noisy digital signal levels are correctly identified with regard to their binary value and can be recreated without the noise of the sampled signal.

Electrical signals are commonly transmitted using a single signal line per information channel between a sender and receiver, whereas a shared return path (usually ground) is used by many channels. This is known as *single-ended* signal transmission (Texas Instruments 2002). Compared to differential signalling, it allows higher integration densities on PCBs and cabling, and is simple to implement and cost effective, but is very prone to noise generation as well as reception. Signal transmission lines implemented as ‘single ended’ emit EM fields when fast, high-bandwidth signal switching occurs, may receive EM fields, and create loops with the return path to form a closed area leading to unwanted voltage induction when exposed to time-varying magnetic fields. Consequently, as electrical analogue signal paths within strong switching MRI gradient and RF fields are prone to induction-related signal distortions (see (i)b above), PET performance is often impaired due to distorted PET timing and energy information during MRI. These effects were reported in Weirich *et al* (2012) and Yoon *et al* (2012) and were even intentionally used for temporal synchronisation between PET and MRT systems (Weissler *et al* 2015b). Therefore, the earlier components and electrical transmission techniques both susceptible to RF-related signal disturbances such as APD/SiPM and any other single-ended-based analogue electrical signalling within the PET signal acquisition path located inside the MRI bore are digitized, the higher the likelihood of maintaining the intrinsic PET signal SNR and thus the PET performance.

However, digitizing earlier in the PET signal chain in this way unfortunately increases disturbances to MRI as described in (ii) because of additional PET electronics located within the MRI field of view (FoV). PCB design, signal transmission types such as ‘single ended’ and frequent signal switching combined with voltage level changes using strong signal slopes, as is the case for analogue SiPM signals or the periodic electrical signal switching associated with digital electronics, all have an impact on EM fields that are emitted by cabling and PCB traces. In this context, the latter act as antennas (Ott 2009). Such EM fields with field patterns and frequencies in the RF range to which an MRI system is sensitive couple into the MRI RF receive chain and deteriorate the MRI SNR and thus MR image quality as reported in Maramraju *et al* (2011), (2012), Wehner *et al* (2014), Weissler *et al* (2014), Yamamoto *et al* (2011) and Yamamoto *et al* (2012). For instance, Yamamoto *et al* (2012) reported an MRI SNR decrease from 350 (MR phantom imaging without SiPM PET) to 200 when their PET ring was operating during MR image acquisition. In Weissler *et al* (2014) an average MRI SNR degradation of 13% was determined for six standard MRI sequences caused by PET-related RF interference. Usually, EM interference (EMI) shielding techniques for PET detector electronics located in or very close to the MRI bore are applied to suppress this unwanted, spurious RF signal coupling (Judenhofer *et al* 2007, Catana *et al* 2008, Olcott *et al* 2009, Peng *et al* 2010, Maramraju *et al* 2012, Yamamoto *et al* 2012, Hong *et al* 2013, Weissler *et al* 2014), which we define in the scope of this paper as noise. As RF shielding always contains conducting materials, eddy currents generated within the shields by switching MRI magnetic fields ((ii)b and (ii)c) may result in MR image artefacts (Hennel 1997, Wehner *et al* 2015). Additionally, RF shielding increases system design complexity and costs and may be susceptible to RF field leakage. Hence, to entirely prevent PET-detector-shield-related eddy currents to preserve MR image quality and to optimise costs and design, a PET detector operated without any shielding at all would be a favourable approach, but would require us to avoid the PET-related RF noise coupling into the MRI RF receive chain. For such a scenario, the RF emission of the detector would need to be reduced and adapted to minimise the RF interferences.

In Gebhardt *et al* (2015), a method was presented to reduce the PET detector’s RF emission by stopping the PET data acquisition during the active MR RF signal receive period. However, the higher the MR RF signal acquisition duty cycle is, the longer the overall PET scan time needs to be in order to account for the same number of recorded PET data used for PET image reconstruction when compared to an uninterrupted PET scan. Depending on the PET–MR image protocol and the radioisotope used, this trade-off between MRI SNR preservation and increased PET scan time might not be acceptable.

In this paper, we present initial investigations of techniques which aim at the modification of the PET electronics’ RF field emission. These techniques are demonstrated by specifically addressing the clocked digital part of the PET electronics. By shifting the clock frequencies and by modifying clock phases to obtain different clock phase patterns between electronic components and/or signal traces, RF field distributions and frequencies emitted by PET detectors can be adjusted to couple as little as possible into the MRI RF coil, thereby reducing the PET-related MRI RF noise.

In principle, these techniques are applicable to any clocked, digital circuits operating within or close to the MRI. This does not only include local PET signal digitization as done by dSiPMs in case of the Hyperion II^D^ insert and analogue SiPMs combined with mixed-signal ASICs performing local digitization as done in the case of the clinical GE SIGNA PET–MR and the Hyperion I insert (Weissler *et al* 2014). It also covers digital electronics used e.g. to control the PET detector and monitor operating and ambient parameters such as voltages and temperatures.

We implemented the RF interference reduction techniques by solely modifying firmware of field-programmable gate arrays (FPGA). The FPGAs’ reconfigurability, flexibility and—in relation to their physical sizes—increasing amount of logic resources, which are of interest for the growing need of versatile data processing capabilities, make them very suitable for highly integrated, compact detector systems. We believe that the reconfigurability applicable to our proposed RF interference reduction methods could extend application-related possibilities of PET–MRI and SPECT–MRI devices thanks to adaptive RF interference reduction. As examples, PET and SPECT detectors built as inserts for combined imaging with MRI could be adapted regarding RF field emission if they are to be used with different MRI systems. Similarly, by reconfiguring the FPGA firmware, the RF emission characteristics of a PET/SPECT detector could be adapted to minimise RF emission with regard to different types of MRI RF coil such as commonly used body, head, breast and knee coils used with the same MRI system. It also allows for further steps towards highly integrated PET detectors within MRI systems to maintain a large, transaxial, bi-modal FoV.

## 2. Materials

To implement, test and study our RF interference reduction techniques, we used one PET module of our Hyperion II^D^ PET insert for simultaneous PET–MRI (Weissler *et al* 2015a). This section first gives an overview of the PET module with a focus on data-acquisition-related parts. It follows a detailed description of the capabilities offered by the clock signal distribution and processing infrastructure that is used for the digital electronics and PET sensors. This will provide important background for the methods and applied investigations to be presented in section 3.

### 2.1. Singles detection module overview

**Figure 1.**
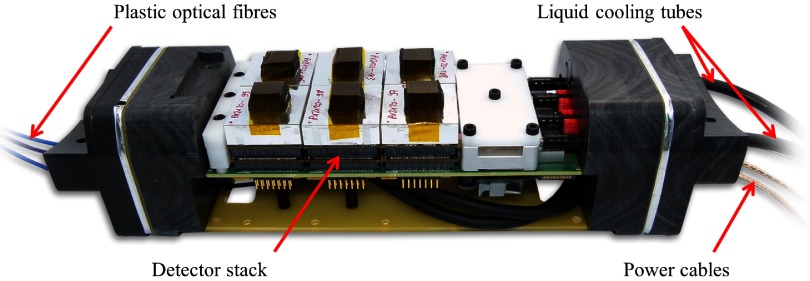
Singles detection module fully equipped with six detector stacks and removed carbon fibre shielding.

**Figure 2.**
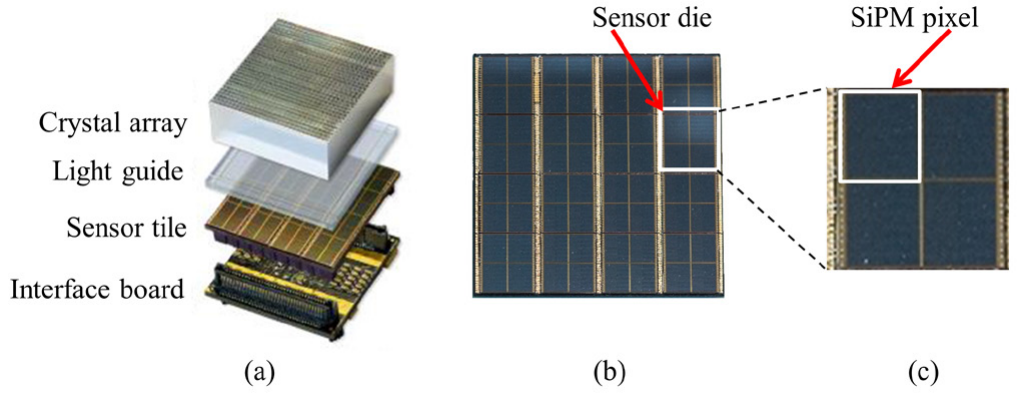
(a) One complete detector stack (exploded view). PCB images taken from Weissler *et al* (2015a). (b) A sensor tile consisting of 16 sensor dies with one of them visualised by a white square. (c) A single sensor die.

A *singles detection module* (SDM) (depicted in figure 1) may include up to six detector stacks (Dueppenbecker *et al* 2012) in a $2\times 3$ stack arrangement. Every stack (shown in figure 2(a)) is composed of a lutetium–yttrium oxyorthosilicate (LYSO) scintillator crystal array with 900 crystals (crystal dimensions: $(1\times 1\times 12)$ m^3^) and a light guide (2 mm thickness) glued between the crystal array and a *sensor tile*. The latter is a PCB equipped with SiPMs on top and connectors on the bottom side to be connected to an *interface board*. One sensor tile comprises 16 Philips DPC 3200-22 digital SiPMs (Degenhardt *et al* 2009, Frach *et al* 2009, 2010) (see figure 2(b)), each of them combining four SiPM pixels with logic for triggering, photon counting and time stamping on a monolithic silicon die (Rabaey *et al* 2002), which is equal to 64 SiPM pixels per detector stack. To clearly differentiate between one logical SiPM pixel and one DPC sensor containing four of these as depicted in figure 2(c), a DPC sensor is also referred to as a *sensor die* for clarification purposes throughout this paper. Each sensor die is typically clocked with a frequency of 200 MHz, offers a bi-directional trigger port to either accept or drive an external trigger signal, uses a JTAG interface (IEEE 2001) for configuration purposes and transmits sensor data via dedicated communication lines at half the sensor die clock frequency. The DPC sensors directly interface to the *data-acquisition-and-control architecture* (DACA) (Gebhardt *et al* 2012) of the Hyperion II^D^ PET insert. Sensor die clocking, configuration, trigger line interfacing and sensor data reception are undertaken individually for each of the 16 sensor dies of a sensor tile by the *stack FPGA* (Xilinx Spartan-6 (www.xilinx.com)) located on the interface board. DPC sensor data received by each stack FPGA is processed and transmitted via point-to-point links to a larger FPGA (Xilinx Virtex-5) of the SDM main board referred to as the *singles processing unit* (SPU) (Weissler *et al* 2012). There, data are further processed, concentrated and forwarded optically to the *data-acquisition-and-processing server* (DAPS) (Goldschmidt *et al* 2013) located outside the MRI room using Gigabit Ethernet and UDP-IP protocols.

### 2.2. Hyperion II^D^ clock signal distribution and processing

**Figure 3.**
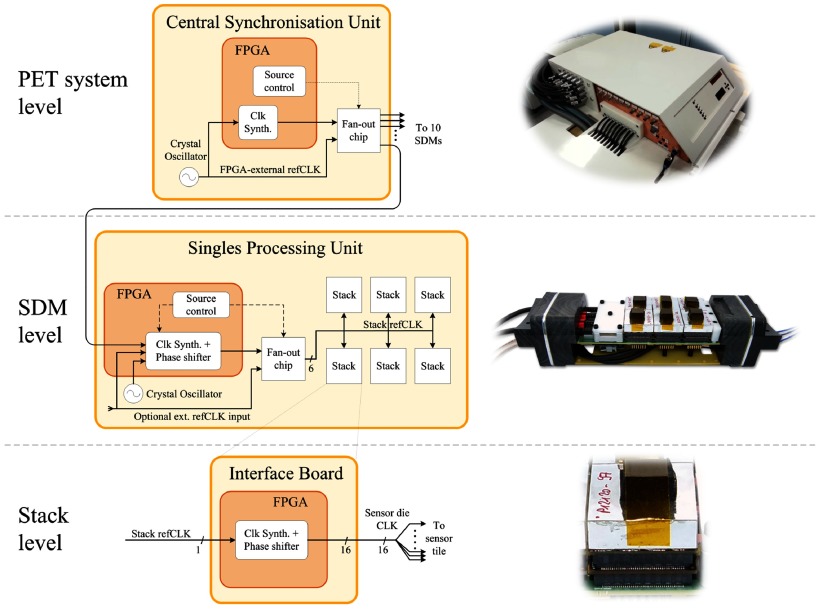
Clocking infrastructure of the PET insert visualised for the reference clock (refCLK) and refCLK-derived clocks at PET system, SDM and stack levels. Arrows with dashed lines indicate clocking-related control signals, arrows with full lines indicate clock signal paths. At SDM level, the number of individual stack refCLK signal lines on the SPU is indicated by the number Six below the signal line close to the fan-out chip. The frequency and phase of the clock signals can be modified in each FPGA via integrated clock synthesizer and phase shifter blocks.

In addition to the optical data communication link, every SDM is equipped with a dedicated optical link to accept external, PET-system-wide-synchronous clock signalling and control pulses from the *central synchronisation unit* (CSU). This clock signal is referred to as the PET system reference clock (refCLK) and sources clock-synthesizer- or clock-divider-derived clocks driving the sensor dies, FPGA PET data processing chains and time stamping units. For research purposes, the CSU and SDMs were designed to allow for versatile refCLK routing and processing capabilities at PCB as well as FPGA firmware levels, which is shown in figure 3. The CSU and the SPU boards make use of a dedicated high-speed, low-jitter and low-skew fan-out chip, which accepts two different clock signal inputs. They are software selectable using the PET insert control software, which configures a firmware module driving the ‘source control’ line of the fan-out chip. On the CSU, this allows us to select either a crystal oscillator as the direct refCLK source or a refCLK signal, which may be modified using an FPGA-internal clock frequency synthesizer and phase shifter on the way to the fan-out chip input. The fan-out chip has the task of forwarding one of the input signals to all SDMs while keeping skew and jitter as low as possible.

The SPU FPGA of an SDM accepts three different refCLK signal inputs: one provided by the CSU, a clock signal source from a local crystal oscillator on the SPU board, or for test purposes a signal via a board connector to be provided from an external source such as a signal generator. Within the SPU FPGA, one of these three clock signals may be chosen via software to serve as the source for an FPGA-internal block offering clock frequency synthesis and phase shift. From there on, the resulting FPGA-internal clock signal or alternatively an FPGA-external signal from the optional SPU connector is forwarded to the detector stacks via the SPU fan-out chip. Although depicted by a single line in the SPU clock signal diagram of figure 3 for simplicity reasons, six individual stack refCLK signal paths sourced by the fan-out chip are used; one per stack.

At stack level, the stack refCLK signal is routed to the stack FPGA on the interface board and drives the input of an FPGA-internal clock frequency synthesizer/phase shifter prior to being forwarded individually to each of the 16 sensor dies on the sensor tile.

With the above described capabilities, the presented clocking infrastructure allows us to vary the refCLK signal frequency and phase at different hierarchy levels of the DACA architecture: Clock modifications may be introduced at PET system level, SDM level or stack level down to the level of individual DPC sensors of each sensor tile while being derived from a common clock source to enable fixed phase and frequency relationships between sensor die clock signals.

From an electrical signal point of view, all refCLK signals are transmitted using the low-voltage differential signalling (LVDS) standard on the CSU and SPUs to increase electromagnetic compatibility (EMC) of the PET insert’s electronics. Only the signalling between sensor dies and stack FPGAs was chosen to be of single-ended type to allow successful routing of all signal traces due to the high signal-lines-to-sensor-tile size ratio.

For research- and development-related applications making use of single SDM setups, the message-based data communication of the DACA conveniently allows SDMs to be directly connected to and operated by a control PC running Hyperion control software without the need for the data acquisition server system as normally used within the standard configuration in conjunction with ten SDMs mounted on the Hyperion II^D^ gantry (Weissler *et al* 2015a).

## 3. Methods

### 3.1. Interference reduction methods overview

#### 3.1.1. Clock phase shift method.

The basic idea of the clock phase shift method is to modify the emitted RF fields by applying clock phase shifts between the clocked components of the PET detector. Since our PET detector consists of a plurality of clocked components emitting RF fields and thus contributing to the effective emitted field, the pattern of the field can be modified due to the superposition principle by applying the aforementioned phase shifts. This way, the emitted RF field pattern, which couples to the MRI RF coil, can be altered and the coupling of the externally applied RF fields can be reduced, thereby resulting in less interference between the PET system and the MRI RF receive chain. Here, the coupling is defined by the coefficients (determined by the overlap integral or coupling integral) of a mode expansion of the externally emitted fields in the mode basis function of the RF coil. A smaller coupling coefficient results in a decreased energy transmission efficiency due to the aggravated excitation of the RF coil’s modes.

#### 3.1.2. Clock frequency shift method.

**Figure 4.**
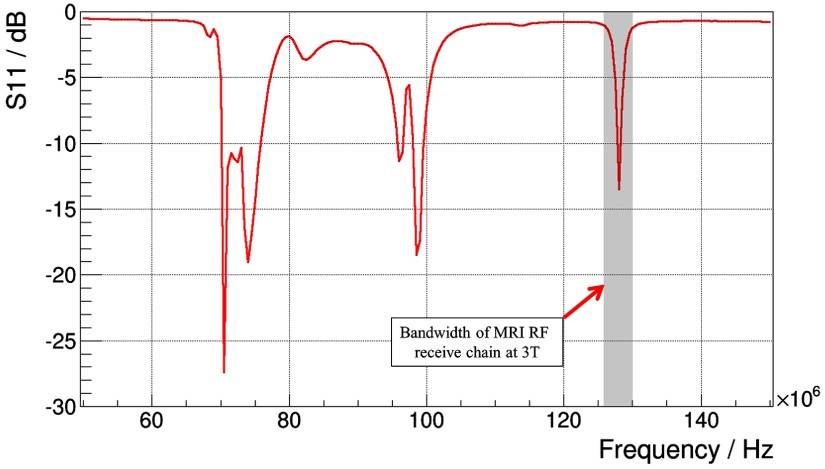
Measured reflection coefficient (S11, magnitude) as a function of frequency for the birdcage resonator. The resonance spectrum includes resonance modes at which an energy transmission to the RF resonator is highly efficient. The mode that provides a homogeneous RF field distribution and is therefore used for spin excitation is highlighted with a grey-shaded area. In contrast, the modes at lower frequencies do not provide a homogeneous field distribution and are therefore not of interest for MRI applications. Hence, a band-pass filter (also indicated by the grey-shaded area) is applied around the Larmor frequency at 3 T. To avoid noise coupling, the sensor die clock frequency is shifted in such a way that as little energy as possible is emitted in the frequency region of the band-pass filter.

Figure 4 depicts the resonance spectrum of our bird cage RF coil, which was measured with a network analyser (Agilent E5071C ENA, USA). It visualises the different resonance modes of the coil. The energy of RF fields with frequencies corresponding to these modes is transmitted with a high efficiency into the coil. The mode which is highlighted with a grey-shaded area provides a homogeneous RF field distribution and is therefore used to generate the *B*_1_ field required for spin excitation. The frequency of this mode is tuned to match the resonance condition (at 3 T:  ∼127.78 MHz). The other modes (visible at lower frequencies), not providing a homogeneous distribution, are not used for MRI acquisition purposes. Therefore, the MRI receive chain includes a band-pass filter around the 127.78 MHz mode to suppress the energy potentially transferred via the lower modes.

The clock frequency shift approach aims at RF interference reduction by shifting the frequencies of PET-detector-related clock signals away from the homogeneous coil resonance mode to reduce EM field amplitudes at the resonance mode frequency and thus reduce the noise coupling into the coil to a minimum.

### 3.2. Simulations of the clock phase shift method

#### 3.2.1. Field emission distribution.

**Figure 5.**
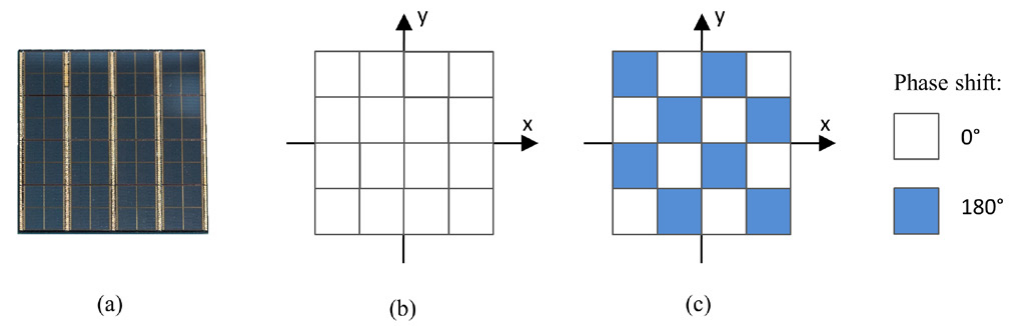
Phase-shift approach at stack level: each of the 16 squares represents a sensor die, which is modelled as a dipole loop. (a) The actual sensor tile with the PET sensor dies as seen from above. (b) The usage of an in-phase pattern. (c) A checkerboard-phase pattern.

In a first step, we analysed this technique regarding its feasibility by implementing a model based on the sensor tile of an SDM: according to the arrangement of the sensor dies on a sensor tile (see figure 5(a)) and since all sensor dies are clocked via single-ended clock lines, we modelled a sensor tile as a system of 16 EM field emitters. As a simplification, each emitter is described as a magnetic dipole sender, resulting in a dipole emitter system with the same spatial arrangement as the sensor tile structure (see figure 5(b)). Based on this model, we calculated the emitted magnetic field distributions using MATLAB in planes parallel to the sensor tile surface to study the compensating superposition effect of the dipole system with clock phase relationships between dipoles that are out of phase (unequal to zero). Here, we focused on two example systems: an in-phase model, in which the phase relation between neighbouring dipoles (sensor dies) is set to ${{0}^{{}^\circ}}$, and a checkerboard model. In the latter, the phase relation between neighbouring dipoles is set to ${{180}^{{}^\circ}}$ (see figure 5(c)).

#### 3.2.2. Field coupling simulation.

**Figure 6.**
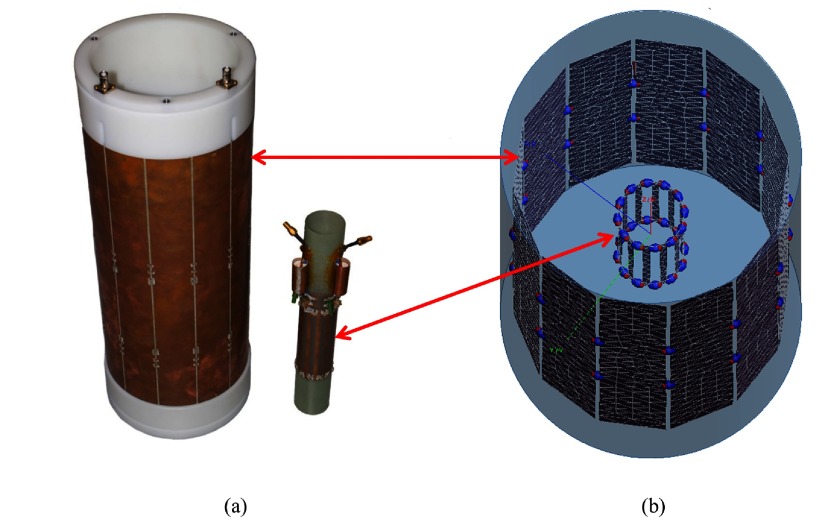
(a) The RF coil screen with the RF coil taken out and placed next to it (image taken from Wehner *et al* (2015)). (b) The FEKO simulation model.

In order to investigate the noise induction originating from external interferences to a birdcage resonator, the field coupling was simulated using a finite element method (FEM) toolkit (FEKO EM Software and Systems (www.feko.info)). Using this software toolkit, we implemented an FEM model of our PET-transparent mouse coil (see figure 6). Both phase pattern models were added via a modelled dipole arrangement in front of the RF screen in the simulation model, and the induced current distribution on the RF resonator was simulated for both phase patterns. To investigate the coupling at an operating point at which the resonator accepts external energy with most efficiency, we set the dipole frequency to the resonance frequency of the simulated RF coil.

### 3.3. Experiments

#### 3.3.1. Sensor die clock frequency.

To investigate the impact of the sensor die clock frequency alternation with regard to resulting RF fields coupled into the MRI RF coil, an SDM was operated at different sensor die clock frequencies in addition to the typical sensor die clock frequency of 200 MHz. First, we chose a frequency equal to the Larmor frequency of the 3 T MRI because we expect the impact regarding RF field coupling into the MRI RF coil to be highest at this frequency, thereby demonstrating the influence of the sensor tile clock frequency related to the RF noise picked up by the MRI. Second, to study noise coupling for a clock frequency within the sensitive bandwidth of the MRI, but with less impact compared to a clock frequency at the Larmor frequency, 140 MHz was added for our measurements. Third, 100 MHz was chosen because its first harmonic is equal to the typical sensor die clock frequency of 200 MHz. Finally, we chose 160 MHz as a candidate located between clocking frequencies in the MRI-sensitive frequency bandwidth and the typical DPC sensor clock frequency.

#### 3.3.2. Sensor die clock phase pattern.

**Figure 7.**
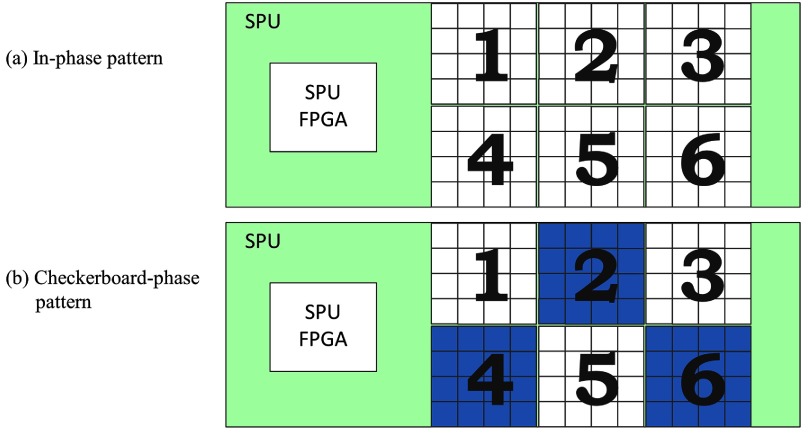
Phase pattern schemes at SDM level for an SDM with six stacks: compared to the default sensor die clocking where no phase shift is applied (a) is a sensor die clock pattern with ${{180}^{{}^\circ}}$ clock phase shifts for complete stacks (stack level) to obtain a checkerboard pattern (b).

The MATLAB-based calculation presented in section 3.2.1 was used to study EM field emission changes upon modifications of the sensor die clock phase at stack level. However, this does not take into account all other electronic components and connectors of the complete stacks as well as the SPU board, which contribute to the overall field emission in addition to single sensor tiles. Therefore, the scope of our measurements to study phase-pattern-related influences was extended by including a complete SDM equipped with six stacks rather than a single detector stack. For the measurements with an SDM, we applied sensor clock phase variations at SDM level, meaning that all sensor dies of a sensor tile are clocked in phase and that a fixed clock phase relation is kept between adjacent sensor tiles of an SDM. To remain consistent with the clock phase pattern chosen for the MATLAB calculation at stack level, measurements with an SDM were performed with a phase shift of ${{180}^{{}^\circ}}$ between adjacent stacks, resulting in a checkerboard pattern as shown in figure 7(b). The measurement results of emitted field amplitudes and field pattern obtained with firmware designs applying clock phase shifts were compared qualitatively to reference measurements where no phase shifts were present.

#### 3.3.3. SDM operation.

As we used a single-SDM configuration for our investigations, the refCLK input signal of the SPU FPGA is not provided by the CSU. Instead, the refCLK signal for the SPU as well as the stack FPGAs is sourced by the 100 MHz oscillator residing on the SPU board. Clock signal frequencies and phases for clocking and data communication between the FPGAs within the SDM are kept identical between the single-SDM operation and the full-PET-ring operation. To obtain the desired clock and phase settings, the firmware design of the stack FPGAs is modified in such a way that the FPGA clock generator (a phase-locked loop) feeding the clock inputs of the sensor dies and clocking the FPGA-internal data processing chain is programmed to our frequency and phase shift needs. These clocking modifications thus only directly affect parts of the stack FPGA’s digital logic and the single-ended signal traces between the interface board and the sensor tile.

The modified FPGA designs undergo synthesis, mapping and routing for the stack FPGA platform using Xilinx ISE tools (Xilinx Inc. (www.xilinx.com)). Afterwards, the resulting bit-file streams are converted into XSVF format. This allows for a stack FPGA configuration via the Hyperion software on the control PC, which communicates with an XSVF-JTAG programmer residing within the SPU FPGA firmware design. This avoids the need to access the SPU board of an SDM with an external JTAG programmer so that the SDM may remain entirely untouched during measurement series using different clock configurations.

To ensure that the PET data acquisition is correctly working with our modified firmware designs, we made singles measurements using one SDM with different firmware designs yielding sensor die clock frequencies and clock phase patterns as given in sections 3.3.1 and 3.3.2 and one ^22^Na source with an activity of 3.95 MBq. The sensor die configuration parameters and operating conditions are set to values we typically use for PET measurements as described in Schug *et al* (2015), section III D. The ‘center-of-gravity (CoG) with adaptive corner extrapolation’ (COG-ACE) algorithm (Schug *et al* 2015) was used to perform off-line PET singles clustering on the acquired raw PET data sets. The firmware design clocking the sensor dies in phase at 200 MHz is the one typically used for the DPC sensors and serves as the reference design.

The SDM operation for all other measurements presented in this paper is as follows. A single SDM is directly connected to a control PC running Hyperion software and all FPGAs of the SDM are configured after SDM power-up. The sensor die configuration settings correspond to those as described above. Afterwards, the data acquisition is launched, during which RF-field-related measurements are made.

### 3.4. Characterisation

To characterise the different clocking schemes in detail, we performed broadband emission scans and field map scans as well as direct test measurements with the MRI system.

#### 3.4.1. Broadband emission scans.

**Figure 8.**
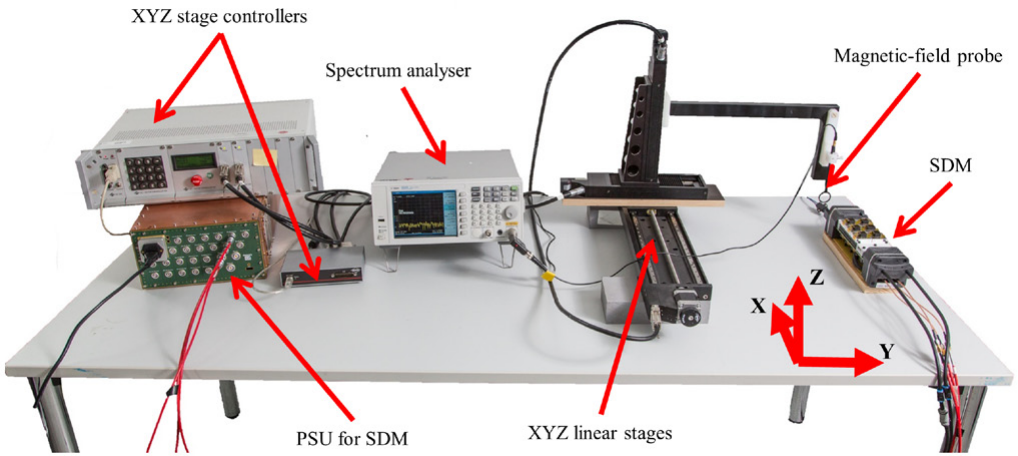
Overview of the laboratory bench setup used for RF field map and RF broadband measurements. Not shown in the picture are the cooling unit and the control PC running Hyperion software, which is connected to the SDM on the laboratory bench.

For a detailed RF field emission characterisation, we used a spectrum analyser (Agilent N9320B, USA) in combination with a magnetic-field probe (Langer EMV, RF-R 400-1, Bannewitz, Germany) which is round-shielded with a diameter of 25 mm. In contrast to measurements with the MRI system, these measurements allow the acquisition of broadband emission spectra to obtain a better overview on the distribution of amplitude magnitudes over the measured frequency range. The different frequency designs were tested by acquiring emission spectra in the range from 50 MHz to 250 MHz. For these measurements, the magnetic-field probe was positioned on the top centre of the SDM and as close as possible to the SDM housing (see figure 8).

#### 3.4.2. Field pattern measurements.

In addition to the amplitude measurement, the magnetic field component of the RF field pattern emitted by an SDM was measured and the result was visualised as a field map covering all magnetic-field probe measurement positions of one plane. We acquired field maps for the *x* and *y* components of the emitted $\vec{H}$ field on planes around the SDM (lateral surfaces of a cuboid) for a frequency range of  ±100 kHz around the sensor die clock frequencies (section 3.3.1). These maps were used to analyse changes in emitted RF field patterns depending on the chosen clock frequency and clock phase patterns and help to locate parts of the SDM which act as strong RF field emitters.

**Figure 9.**
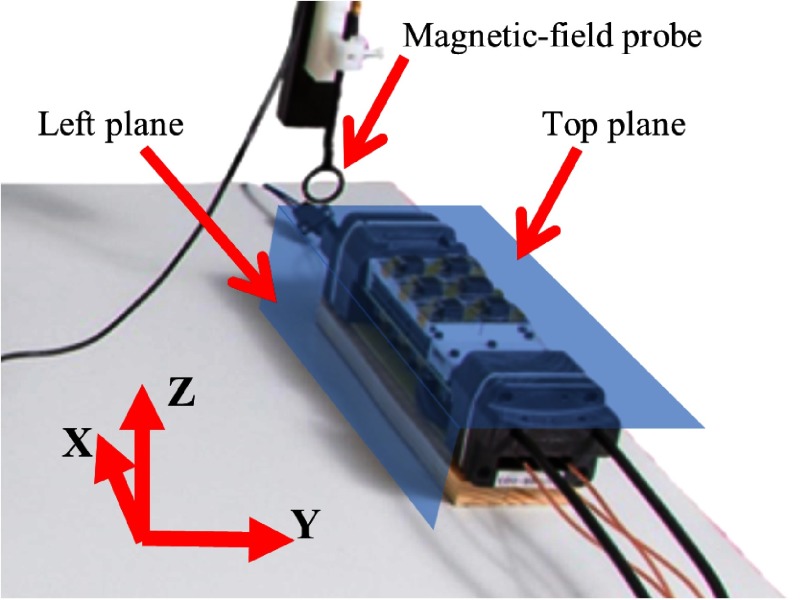
Closeup view of the SDM positioned on the laboratory bench setup as seen in figure 8: the XYZ table moves the magnetic-field probe along the left and top planes to cover in-plane positions with a 5 mm step. Afterwards, the SDM is turned with the detector stacks in the direction of the laboratory bench to repeat the measurement.

The magnetic-field probe was attached to an XYZ table (OWIS, Germany) enabling positioning in 3D space around the SDM under test and thus the acquisition of RF field patterns as shown in figure 8. All cuboid planes were covered by the probe by first measuring the top and left planes of the SDM (see figure 9) and then turning the SDM through ${{180}^{{}^\circ}}$ to repeat the measurement with the left plane becoming the right one and the top plane becoming the bottom one to complete the cuboid. The probe was moved 35 cm along the *x*, 10 cm along the *y*, and 9 cm along the *z* axis, thereby covering 2769 measurement points with a step of 5 mm. As the SDM was turned through ${{180}^{{}^\circ}}$ to measure the field pattern in the other two lateral planes of the SDM, the total number of measurement points is 5539, covering all four lateral planes of the SDM.

#### 3.4.3. MRI noise scans.

**Figure 10.**
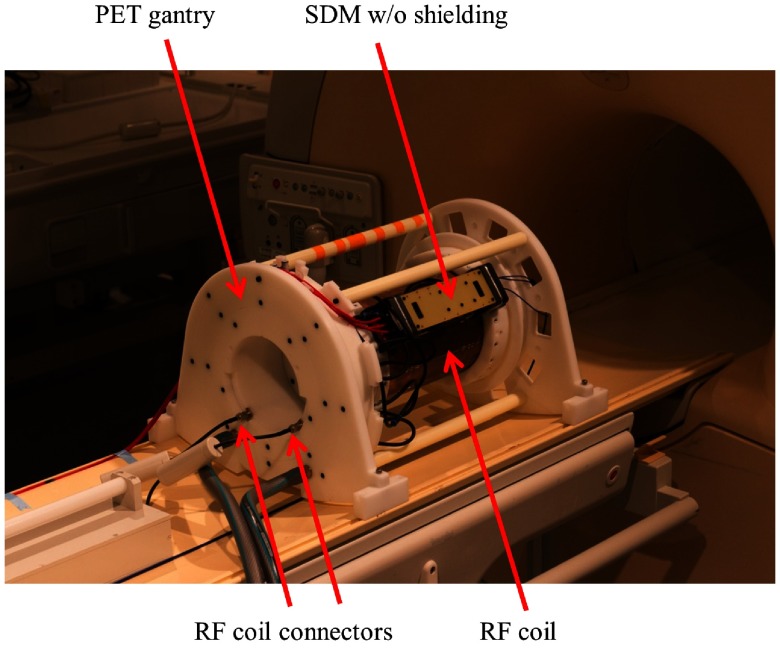
A single SDM without carbon fibre shielding mounted on a PET gantry to make MRI noise measurements. The RF coil was connected via coil electronics to the MRI RF subsystem.

We used a clinical MRI scanner (Achieva 3.0 T, Philips, The Netherlands) to evaluate the implemented frequency and phase pattern modifications with the full MRI acquisition chain (see figure 10). The carbon fibre shielding was removed to allow for larger differences in coupled RF noise for reasons of clarification between different measurement results. To evaluate the RF field emission of an SDM for different sensor die clock frequency and phase scenarios with regard to RF noise coupling into the MRI RF receive chain, dedicated spurious noise scans with the MRI and a PET-transparent Tx–Rx mouse coil (12-leg birdcage resonator, high pass) (Weissler *et al* 2015a) were performed. These MRI scans allow us to quantify the noise signal amplitudes as a function of frequency in the frequency region to which the MRI RF receive chain is sensitive. The measurement protocol is based on sequences provided by the MRI vendor and consists of five turbo-spin-echo (TSE) sequences (${{T}_{\text{R}}}$/${{T}_{\text{E}}}$ 1044/256 ms, turbo factor (echo train length) 32, acquisition matrix $1024\times 1024$, bandwidth per pixel 180 Hz) executed with shifted frequency ranges to cover a total continuous bandwidth of 1 MHz with the Larmor frequency as the centre frequency. As the vendor-provided MRI protocol includes RF pulse excitation (flip angle ${{1}^{{}^\circ}}$), the protocol was modified to exclude any MRI RF excitation so that noise measurement results are not affected by the presence of any subjects such as MRI phantoms in the MRI FoV, which provide RF signal due to MRI RF excitation (Wehner *et al* 2015). For these studies, the SDM was positioned on the most sensitive PET gantry SDM location regarding PET-related noise coupling into the RF coil. The location was determined by performing a noise scan using the SDM with the reference FPGA design (sensor die clock frequency 200 MHz) for all ten SDM positions of the gantry.

#### 3.4.4. MRI SNR measurements.

The impact of the DPC sensor clock frequency variation on MRI was visualised by scanning a cylindrical phantom containing 20 ml of a CuSO_4_ solution within the Tx–Rx mouse coil. We prepared a 2D TSE sequence (${{T}_{\text{R}}}/{{T}_{\text{E}}}=400/157.46$ ms, flip angle ${{90}^{{}^\circ}}$, turbo factor (echo train length) 12, voxel size  =  $0.07\times 0.07\times 0.5~\text{m}{{\text{m}}^{3}}$, number of slices 1, FoV size $80\times 80~\text{m}{{\text{m}}^{2}}$, acquisition matrix $1144\times 1144$, reconstruction matrix $1600\times 1600$) aiming at very high-resolution MR images with low SNR in order to visualise SNR differences. The SDM was again mounted without RF shielding on the gantry at the SDM position where the PET-related noise coupling to the RF coil was determined to be most sensitive and MRI scans were performed. Signal intensity values were determined along a horizontal line profile with a line width of 50 pixels, which crossed the phantom at its centre. Afterwards, the signal intensity values were normalised to the mean intensity at the phantom position and intensity values were averaged over ten histogram bins. With the plots normalised to the phantom signal intensity, the mean values of the noise level for each plot were calculated to determine changes in noise floor related to the SDM operation with different sensor die clock frequencies.

## 4. Results

### 4.1. Simulations of the clock phase shift method

**Figure 11.**
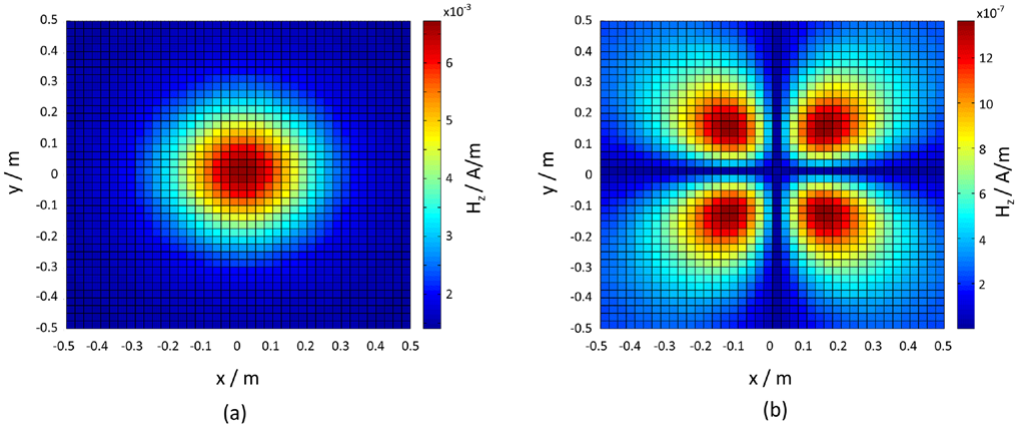
Calculated field emissions of a sensor tile given at a distance of 35 cm: (a) the result for applied in-phase pattern clocking with amplitudes with an order of magnitude of  −3; (b) a quadrupole-arranged field emission when checkerboard-pattern clocking is applied. The amplitudes’ order of magnitude in (b) is  −7.

When simulating the superposed field emission as generated by the 16 sensor dies modelled as magnetic dipoles, the field distribution yielded one large dipole as shown in figure 11(a) in the case of applied in-phase clock pattern. The dipole peak amplitude is located in the centre of the sensor tile. Applying the checkerboard-phase-pattern clocking approach led to emission of a quadrupole field (see figure 11(b)) with peak amplitudes lowered by four orders of magnitude compared to the single peak amplitude generated by the in-phase clocking approach.

**Figure 12.**
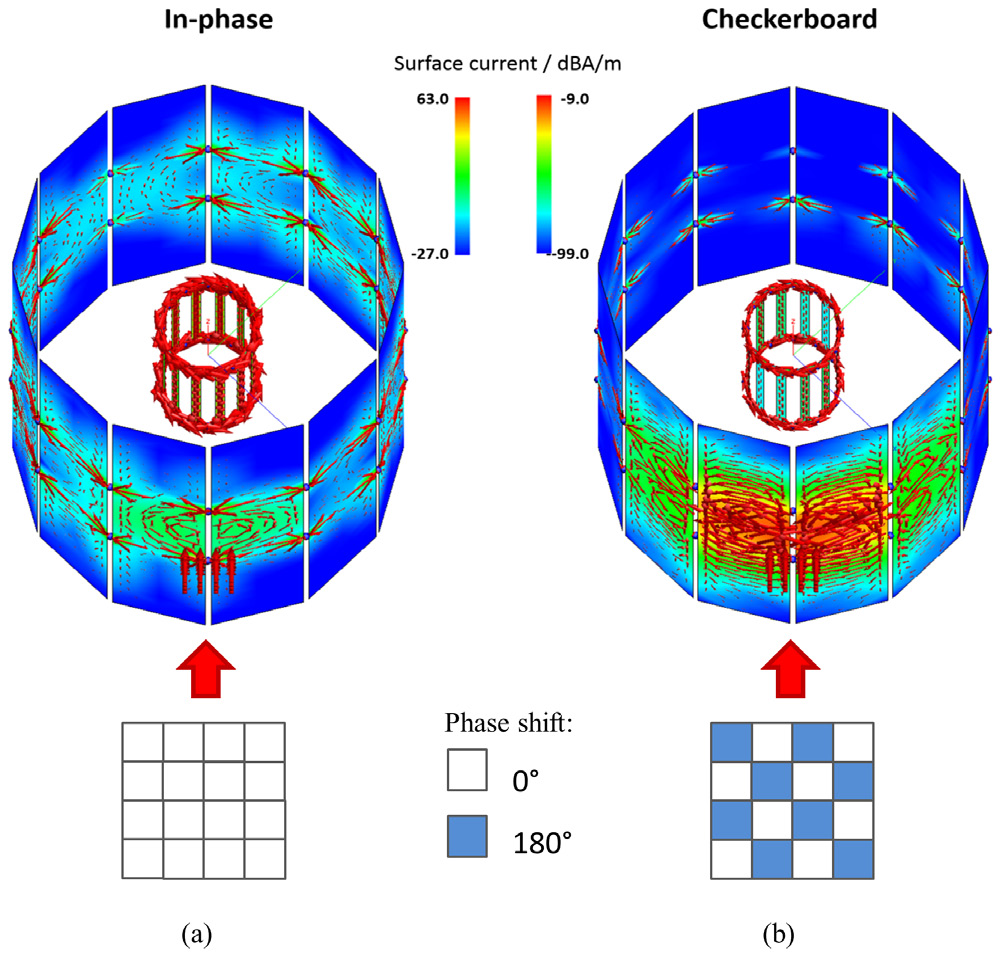
Simulated surface current distributions of the RF screen and the coil resulting from field emission by dipoles (visualised as red arrows) in front of the RF screen. (a) Field pattern generated by the in-phase pattern leads to distribution over entire the RF screen. (b) Checkerboard-pattern-related field emission yields a localised current distribution.

Figure 12 depicts the FEM simulation results of the RF screen and resonator when they are excited by RF field patterns which originate from 16 dipole emitters representing the sensor dies of a sensor tile. The dipoles are represented by the red arrows in front of the RF screen. Applying the in-phase pattern yields a current distribution over the entire RF screen which, in turn, creates RF fields to be coupled with high efficiency into the RF coil (see figure 12(a)). In other words, the RF field pattern created by the dipoles results in unwanted noise, which is strongly coupled into the RF resonator. In figure 12(b) is shown the result when the checkerboard pattern is used. Due to the compensational effect leading to a different field pattern (here of quadrupole type) and reduced peak amplitudes, only a localised current distribution with little overall excitation of the resonator is created. Therefore, the signal coupling into the RF resonator is greatly reduced, as the amplitude of the induced surface currents is reduced. The difference in highest current distribution amplitudes at red spots in figure 12(b) compared to figure 12(a) is equal to  −54 dBA m^−1^.

### 4.2. Experiments

#### 4.2.1. SDM operation.

**Figure 13.**
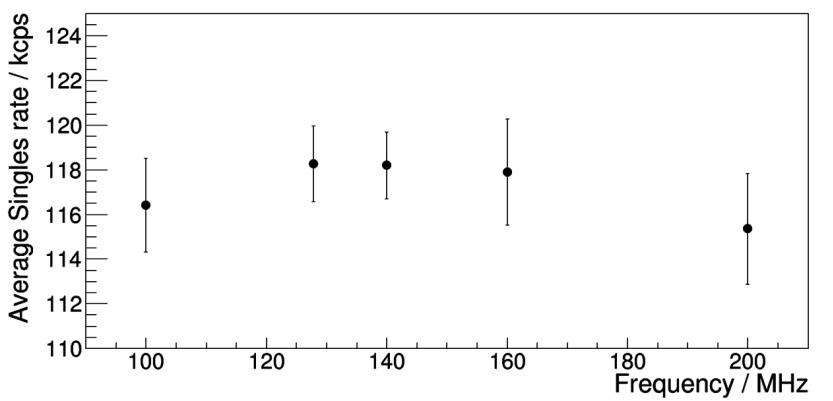
Mean singles rates obtained with firmware designs implementing different sensor die frequencies as given in section 3.3.1. The error bars are given as standard deviations.

All stack firmware designs generating sensor die clock frequencies different from the typically used frequency of 200 MHz (with all dies clocked in phase) as described in section 3.3.1 were successfully tested with regard to expected clock frequency and phase relationships using an oscilloscope (Tektronix MSO4104, USA). In figure 13 are shown the mean singles rates for firmware designs yielding the sensor die clock frequencies described in section 3.3.1. They vary between 115.4 and 118.3 kcps. The mean singles rates for the cases of in-phase and checkerboard-phase patterns at the same clock frequency of 200 MHz are 119.7 kcps (standard deviation (STDd) 2.6 kcps) and 120.1 kcps (STDd 3.2 kcps), respectively.

### 4.3. Characterisation

#### 4.3.1. Broadband emission scans.

**Figure 14.**
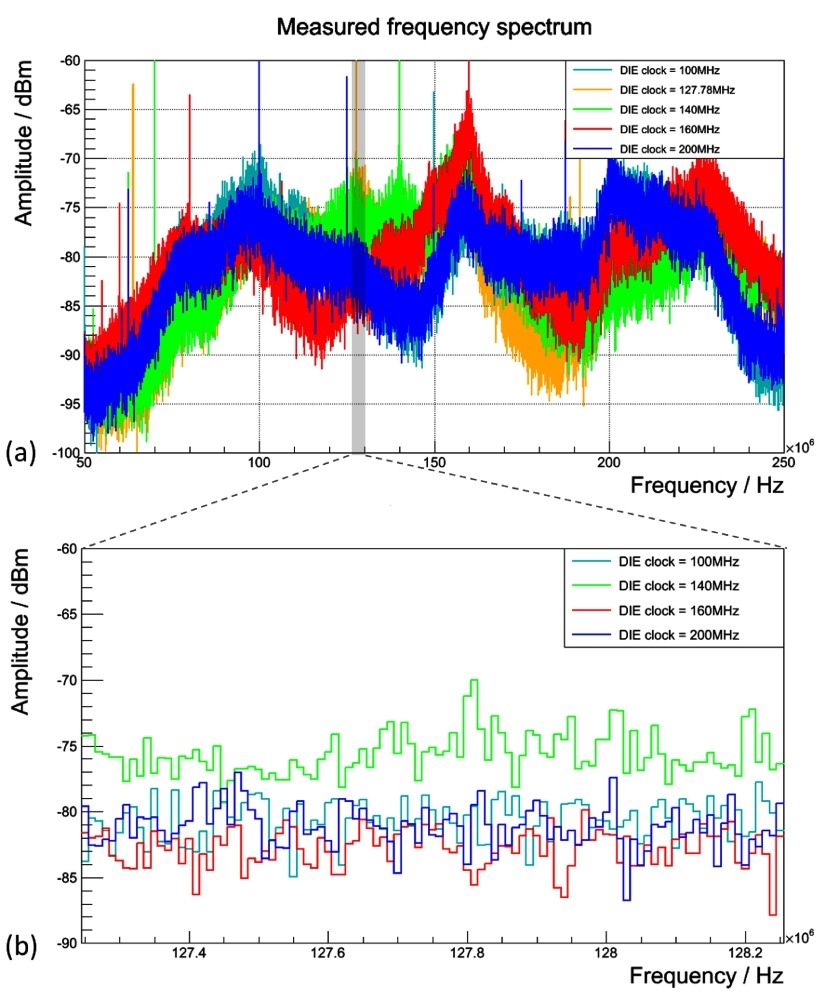
EM emission of the SDM measured with a spectrum analyser for different sensor die clock frequencies. (a) The broadband measurement with the sensitive bandwidth of the 3 T MRI highlighted by the grey-shaded area around 127.78 MHz. (b) The laboratory bench results for a frequency range within the 3 T-MRI-sensitive bandwidth excluding the die clock frequency equal to the Larmor frequency for the worst-case scenario.

Figure 14(a) shows the results of the EM emission measurements made with the laboratory bench setup using the spectrum analyser for all tested sensor die clock frequencies. It is clearly visible that the modification of the die clock frequency enables an alternation of the emitted spectrum. With respect to the sensitive bandwidth of the MRI receive chain (depicted by the grey-shaded bar in figure 14), the die clock frequency resulting in lowest amplitudes around the Larmor frequency is the one at 160 MHz (red), followed by the one at 100 MHz (light blue). Aside from the intentioned worst-case scenario using a die clock frequency of 127.78 MHz (yellow), equal to the Larmor frequency, the worst performance in terms of highest amplitudes within the MRI-sensitive bandwidth is observed with the design clocking the dies at 140 MHz (green). The RF emission distribution is shown in more detail in figure 14(b) for the bandwidth of the 3 T MRI as covered by MRI noise scans for an bandwidth of 1 MHz around the Larmor frequency as described in section 3.4.3. The worst-case scenario was not included in figure 14(b) to better depict the spectrum amplitude differences between the sensor die frequencies different from the Larmor frequency of the 3 T MRI system.

#### 4.3.2. Field pattern measurements.

**Figure 15.**
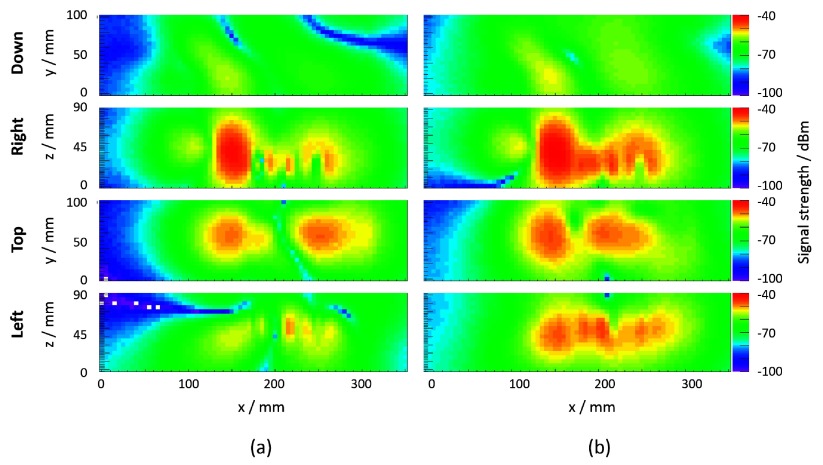
Field map measurement results (*x* component of the emitted *H* field) for sensor die clocking with in-phase pattern (a) and checkerboard-phase pattern (b) at SDM level and a sensor die clock frequency of 127.78 MHz.

**Figure 16.**
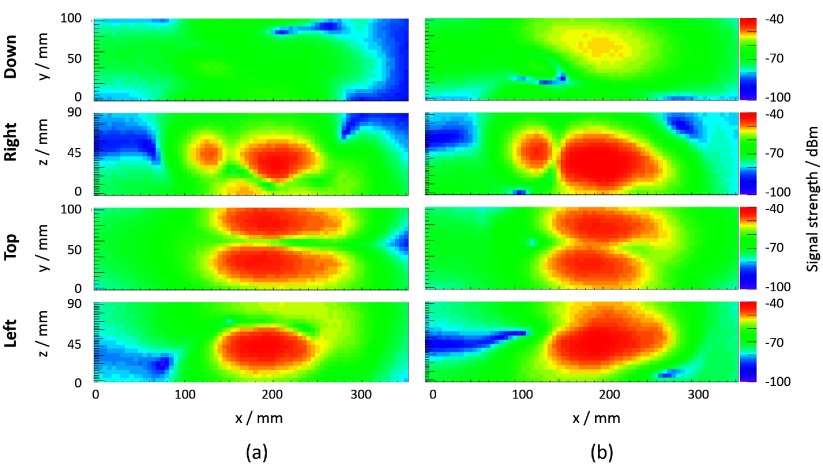
Field map measurement results (*y* component of the emitted *H* field) for sensor die clocking with in-phase pattern (a) and checkerboard-phase pattern (b) at SDM level and a sensor die clock frequency of 127.78 MHz.

The measurement results of the RF-field-related magnetic field component obtained for an in-phase and a checkerboard-phase pattern at SDM level and a sensor die clock frequency of 127.78 MHz are shown in figure 15 (*x* component of the emitted *H* field) and figure 16 (*y* component of the emitted *H* field). In both figures, the four planes of a measured *H*-field component are the following from top to bottom: bottom, right, top and left sides of the SDM. The application of a checkerboard-phase pattern leads to changes in the emitted magnetic field pattern and amplitudes when comparing the *H*-field component H_*x*_ as well as H_*y*_ between the two clock phase patterns.

#### 4.3.3. MRI noise scans.

**Figure 17.**
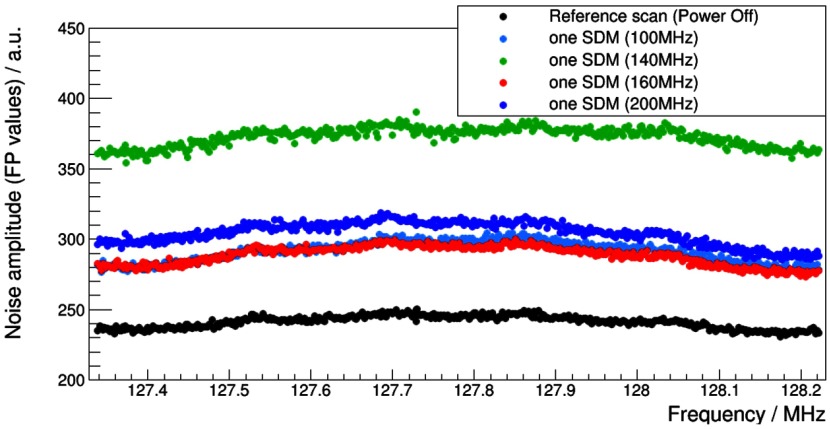
Noise scan results performed for sensor die clock frequencies between 100 and 200 MHz. The SDM was powered off during the noise reference scan.

The noise scan results obtained with the MRI using different sensor die clock frequencies are summarised in figure 17. Least noise was coupled into the MRI receive chain when the PET data acquisition chain of the SDM was operated with clock frequencies of 100 MHz and 160 MHz. A frequency of 140 MHz led to the worst case out of all chosen frequencies. In addition to the noise floor being raised to 350 floating point (FP) values, a distribution of distinct FP measurement points is spread between 350 and 460 FP values. In figure 17, the measurement results marked with black circles represent the MRI noise floor measured with a powered-off SDM.

**Figure 18.**
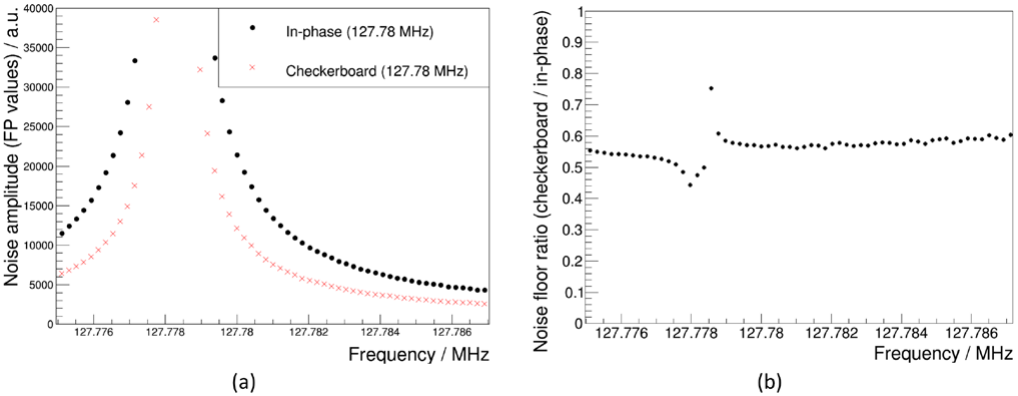
(a) MRI noise scan results for an SDM clocked at 127.78 MHz in-phase and with applied checkerboard pattern at SDM level. (b) Ratio of the noise floors shown in (a).

The noise measurement comparison between in-phase pattern and checkerboard-pattern clocking is depicted in figure 18(a). Both measurements resulted in Lorentzian line shapes due to the clock frequency being at the Larmor frequency at 3 T. The noise floor ratio (see figure 18(b)) for the frequency range shown in figure 18(a) is in the range of 0.5–0.6, meaning that the noise generated with the checkerboard pattern is reduced by almost a factor of two compared to the in-phase pattern.

#### 4.3.4. MRI SNR measurements.

**Figure 19.**
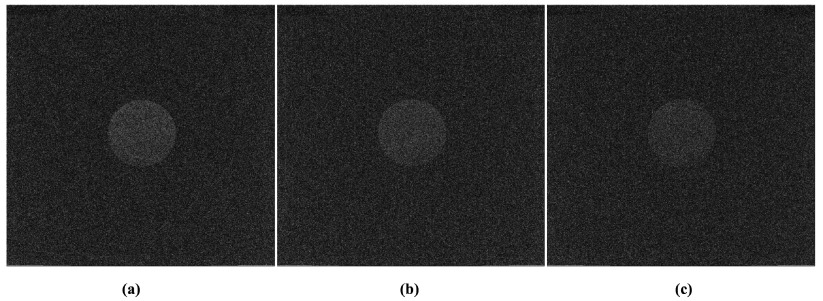
MRI phantom images with low SNR acquired while (a) the SDM is powered off, (b) the SDM operates with a sensor die clock frequency of 160 and (c) the SDM operates with sensor dies clocked at 140 MHz.

In figure 19 are shown three MR images which were acquired while the SDM was powered off (reference scan, image figure 19(a)), while the SDM was acquiring data with the DPC sensors clocked at 160 MHz (image figure 19(b)) and during SDM data acquisition with a sensor die clock frequency of 140 MHz (image figure 19(c)). The image quality is deteriorated in image (b) compared to (a) and is worst in image (c).

**Figure 20.**
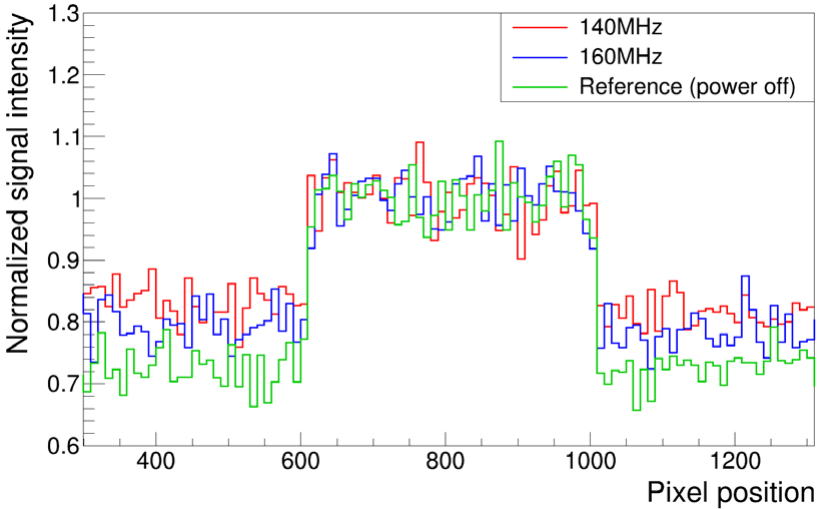
Signal intensities across a horizontal line profile crossing the MRI phantom: The noise floor varies depending on the sensor die clock frequency generated by the SDM firmware.

The clock-frequency-dependent impact on the noise floor is distinguishable via the line profile determined for the three different MR images as shown in figure 20. The noise floor is lowest for the reference scan (noise mean value $0.722\pm 0.004$, SNR 1.385). When the SDM is in acquisition mode, the lowest noise floor (mean value $0.800\pm 0.004$, SNR 1.25) is obtained using a DPC sensor clock frequency of 160 MHz. The highest increase in noise resulting in lowest SNR occurs when the SDM clocks the sensor dies at 140 MHz (noise mean value $0.839\pm 0.004$, SNR 1.192). The relative difference with respect to the noise floor measured for 160 MHz is 4.88%.

## 5. Discussion

### 5.1. Simulations of the clock phase shift method

In principle, RF fields emitted by electronics can be altered with regard to amplitude and spatial distribution by e.g. adapting the signal trace layout on PCBs, choosing electrical signal transmission standards such as LVDS and adapting the frequency of digital, clocked components and traces. Furthermore, the RF field emission may be altered and controlled (to a limited extent) if a plurality of a clocked, digital component type such as the DPC sensor can be identified within electronics and if clock phase shifting can be applied to such components. In our case, we illustrated this principle by calculating a model of the *H*-field distribution motivated by the geometry and electrical signal properties of our sensor tile. We modelled the sensors as magnetic dipoles which oscillate in opposite directions, thereby representing sensor dies clocked with a phase shift of ${{180}^{{}^\circ}}$ compared to adjacent DPC sensors on a sensor tile. This yielded a change of the emitted RF field distribution and field amplitudes compared to the scenario where all sensors are clocked in phase. As the signal induced in the MRI RF coil depends on the coupling efficiency between RF fields and the RF coil, a modification of clock phases with a distribution different to the typical in-phase scenario can be used to control the spurious signal (noise) induction in the RF coil. These principles were demonstrated by performing FEM simulations taking into account our RF shimming, coil geometry and its electrical specifications, and an *H*-field excitation with field patterns as obtained by our MATLAB calculation for a sensor tile. The RF resonator signal intensities determined at the coil were reduced by about five orders of magnitude for the FEM simulation with the checkerboard-phase pattern at sensor tile level compared to the simulation using the in-phase pattern. These simulations are based on a simplified model of the sensor tile and do not cover all details of the actual physical RF emission characteristics. Hence, our presented simulations serve as a proof of principle.

### 5.2. Experiments

#### 5.2.1. SDM operation.

As shown by the results in figure 13, the SDM successfully acquired PET data even with our firmware modifications with regard to different clocking distribution patterns by means of sensor die clock frequency and phase shifts. However, a change of the clock frequency at which the DPC sensor is operated requires us to revise sensor-specific parameter settings which are clock-frequency dependent if aiming to keep values with regard to absolute time. Moreover, the lower the operating frequency of the sensor die, the higher the dead-time properties of the sensor. Dead-time in this context does not only refer to the amount of time needed to be able to successfully detect two successive scintillation events. It also covers the DPC sensor’s bandwidth limitation, which decreases with decreasing operating frequency. The frequency dependence is related to the digital, clocked logic of the main acquisition controller of the DPC sensor (Frach *et al* 2009, 2010). The deterministic behaviour of the finite state machines in the acquisition controller, on the other hand, allows us to predict the frequency-dependent maximum bandwidth limitation per DPC sensor and thus the count rate limitations by calculations and simulations. Summarised, it can be derived that an MRI noise reduction obtained via PET-data-acquisition-related frequency adaptations may result in a trade-off between the gained noise reduction on one hand and the PET detector dead-time on the other hand, provided that the frequency adaptations affect clock domains such as the dSiPM operation or digital hardware- and firmware-based PET data acquisition.

### 5.3. Characterisation

#### 5.3.1. Clock frequency shifts.

Since RF resonators are designed to have characteristic resonance spectra (figure 4), the EM field coupling efficiency between a resonator and EM field transmitters strongly depends on the frequency of the field generated by external sources. As the spectrum measurements in figure 14(a) demonstrate, the spectrum of the EM field emission can be successfully altered by frequency modifications of the sensor die clock signal. Clock frequencies which fall within the bandwidth to which the MRI receive chain is sensitive will inevitably lead to a strongly increased noise floor due to the high amplitudes of the first harmonics of the periodic clock signal. This was demonstrated for the clock frequency of 127.78 MHz in figures 14(a) and 18(a). In addition to distinct amplitude peaks visible in the measured broadband spectra, a noise floor covering a continuous frequency range (in the frequency domain) is usually a result of an overlap of many EM field sources such as non-periodic electrical signal changes in components as well as PCB traces and connectors. The spectrum measured during SDM operation with a DPC sensor frequency of 140 MHz serves as a good example to demonstrate that, although this frequency does not fall directly within the MRI acquisition bandwidth ($128~\text{MHz}\pm 500$ kHz when performing noise scans), this clock frequency leads to a considerable noise floor rise around the Larmor frequency at 3 T, as visualised by the green plot in figures 14(a) and (b). This noise floor increase may be caused by non-periodic-data-line-switching-related field emission, whose spectra are influenced by the choice of the sensor die clock frequency while overlapping with RF fields originating from electronics not affected in their radiation behaviour by the sensor die clock frequency. Choosing a suitable clock frequency to lower the amplitudes of irradiated spectra within the MRI-sensitive bandwidth is therefore not trivial and in the end requires us to make spectrum measurements with analyser equipment and spurious noise measurements with the MRI and the desired RF coil setup. Our results obtained with frequencies of 100 MHz and 160 MHz clearly demonstrate that well chosen frequencies help to reduce PET-related RF noise, or in other words increase the RF silence of our PET detector.

The low MRI SNR obtained with the MR sequence is visible in the reference scan shown in figure 19(a). Counts detected by the SDM were created by low LYSO background radiation and dark counts related to thermal excitation. Except for the singles measurements, this corresponds to the measurement conditions in the laboratory and for the MR noise scans where no radioactive source was used. Placing a source in the vicinity of the SDM to change signal switching behaviour due to increased PET data message transmission between the stack and the SPU FPGAs may modify the RF field emission in such a way that the noise coupling is increased to further illustrate differences in MRI SNR deterioration at a higher order of magnitude depending on the chosen clock frequency. This, however, was not possible as no radioactive sources were allowed in the MRI facility where the SNR measurements took place. Nevertheless, the lower noise floor measured with an SDM operating with a clock frequency of 160 MHz compared to one with 140 MHz demonstrates that clock frequency adjustments of digital circuits can reduce the noise coupled into the RF coil. We expect the differences in terms of visual artefacts in MR images for a broader range of sequences to be more pronounced for the case of a measurement setup scaled up to ten SDMs (a complete PET insert) and using a coil such as a birdcage rabbit coil (presented in Weissler *et al* (2015a)) located closer to the PET detector than the mouse coil.

#### 5.3.2. Clock phase pattern changes.

Our FEM simulations demonstrated that field pattern modifications can principally result in a reduction of RF field coupling into a RF resonator. These modifications were implemented and tested with an SDM by adapting the DPC sensor clock in-phase pattern to a checkerboard-phase pattern at SDM level. Comparing the characterised field emission maps depicted in figures 15 and 16 yields the conclusion that the emitted field distribution was modified upon a change of the clock phase pattern. In particular, the comparison of the *top* plane (which is oriented towards the PET module) measurements shows shifted peaks with similar peak amplitudes. Based on the simulation result, this alteration of the emitted field pattern is expected to change the coupling coefficient to the RF coil and thus is expected to show a different spurious signal (noise) production. This is indeed the case, as shown by the MRI noise measurements (figure 18).

## 6. Conclusion

For imaging systems aiming at simultaneous PET–MRI, the earlier the PET detector’s electronic signals representing PET information are digitized along the PET data acquisition chain residing within the MRI, the higher the likelihood is of avoiding PET signal degradation during MRI RF excitation to preserve the intrinsic PET signal SNR. The drawback is a potential increase of RF field emission caused by digital, clocked electronics within the MRI environment. This leads to an increase of PET-related spurious signal (noise) coupling into the MRI RF chain, which results in an MRI SNR decrease and thus image quality degradation. A conventional way to overcome this problem is the use of shielding materials to suppress PET-related RF field emission. However, shielding introduces the generation of eddy currents, which locally disturb the homogeneity of the MRI B_0_ and gradient fields, increasing costs and mechanical design complexity.

We have presented in this paper two methods to reduce PET-related noise coupling into the MRI RF receive chain which are unrelated to PET detector shielding techniques. Instead of suppressing RF fields emitted by PET, we influenced their generation by modifying the frequency and phase of the clock signal driving the DPC sensors in our Hyperion II^D^ PET insert. The high flexibility of our FPGA-based PET architecture enables clock frequency and phase modifications via firmware programming, which allows easy adaptations to demonstrate our proof-of-concepts. As a first method, we shifted DPC sensor clock frequencies to values different from the typically used one of 200 MHz. The second method treating clock phase modifications was investigated by simulations as well as measurements.

The measurement results using a single SDM demonstrated that our methods principally influence the spectra and distribution of the emitted RF fields with the aim of obtaining RF interference reductions towards the MRI. Concluding, an optimised RF field emission yielding reduced MRI noise coupling helps to mitigate PET detector RF shielding requirements and can allow for a reduction of MRI gradient field distortions close to PET detectors by using less or no RF shielding. Additionally, we believe that these methods have a great potential to support future PET detectors to be used in MRI systems of different field strengths or for one MRI system with different RF coil configurations. FPGA-based clock frequency adaptations applied in the field via software-based configuration choices could be performed and increase the PET–MRI performance and the imaging application versatility. The methods presented in this paper are not restricted to the DPC sensor and our PET insert. They can be applied regardless of the detector technology wherever digital electronics are used either near or within the MRI bore to optimise RF field emission in order to reduce PET-related RF interferences.
